# Mulberry Leaf and Neochlorogenic Acid Alleviates Glucolipotoxicity-Induced Oxidative Stress and Inhibits Proliferation/Migration via Downregulating Ras and FAK Signaling Pathway in Vascular Smooth Muscle Cell

**DOI:** 10.3390/nu14153006

**Published:** 2022-07-22

**Authors:** Tsung-Yuan Yang, Yi-Liang Wu, Meng-Hsun Yu, Tung-Wei Hung, Kuei-Chuan Chan, Chau-Jong Wang

**Affiliations:** 1 Department of Internal Medicine, Chung-Shan Medical University Hospital, No. 110, Section 1, Jianguo N. Road, Taichung 402, Taiwan; marven923@gmail.com; 2 School of Medicine, Institute of Medicine, Chung-Shan Medical University, No. 110, Section 1, Jianguo N. Road, Taichung 402, Taiwan; a6152000@ms34.hinet.net; 3 Division of Cardiovascular Surgery, Chung Shan Medical University Hospital, No. 110, Section 1, Jianguo N. Road, Taichung 402, Taiwan; cvs156@gmail.com; 4 Department of Surgery, School of Medicine, Chung-Shan Medical University, No. 110, Section, Jianguo N. Road, Taichung 402, Taiwan; 5 Institute of Medicine, School of Medicine, Chung Shan Medical University, No. 110, Section 1, Jianguo N. Road, Taichung 402, Taiwan; ya780522@gmail.com; 6 Department of Health Industry Technology Management, Chung Shan Medical University, No. 110, Section 1, Jianguo N. Road, Taichung 402, Taiwan; 7 Department of Medicine, Division of Nephrology, Chung Shan Medical University Hospital, Taichung 402, Taiwan; 8 Department of Medical Research, Chung Shan Medical University Hospital, No. 110, Section 1, Jianguo N. Road, Taichung 402, Taiwan

**Keywords:** neochlorogenic acid, diabetic atherosclerosis, FAK signals, mulberry leaf extract, VSMC migration and proliferation

## Abstract

Mulberry leaf (*Morus alba* L.) has been used as a health food and in traditional medicine to treat several metabolic diseases, including diabetes, hypertension, and hyperlipidemia. However, the mechanism by which mulberry leaf and its functional components mediate atherosclerosis remains unclear. This study aimed to determine the effect of mulberry leaf extract (MLE) and its major component, neochlorogenic acid (nCGA), on the proliferation and migration of rat aortic vascular smooth muscle cells (VSMCs, A7r5 cell line) under diabetic cultured conditions (oleic acid and high glucose, OH). Our findings showed that MLE and nCGA significantly inhibited cell proliferation and migration in A7r5 cells as determined by a scratch wound assay and a Transwell assay. Furthermore, we observed MLE and nCGA inhibited cell proliferation and migration, such as reducing the phosphoinositide 3-kinases (PI3K)/protein kinase B (Akt), focal adhesion kinase (FAK), and small GTPase proteins using Western blot analysis. In conclusion, we confirmed the anti-atherosclerotic effects of MLE and nCGA in reducing vascular smooth muscle cell (VSMC) migration and proliferation under diabetic cultured conditions via inhibition of FAK/small GTPase proteins, PI3K/Akt, and Ras-related signaling.

## 1. Introduction

Metabolic syndrome is a combination of obesity, dyslipidemia, diabetes, and hypertension and is associated with chronic diseases, such as cardiovascular sequelae and atherosclerosis [1,2]. Metabolic impairment causes lipid peroxidation as well as an increase in oxidative stress, vascular inflammation and endothelial dysfunction [3]. This further promotes the oxidative modification of low-density lipoprotein (LDL) particles, which migrate into the arterial wall during the process of foam cell formation. These foam cells secrete several growth factors to facilitate the proliferation of VSMC, leading to fatty streaks and plaque formation [4]. Previous studies have reported that abnormal proliferation and migration of VSMCs from the arterial middle layer to the endothelium is a key process that accelerates atherosclerosis in diabetes [5]. Therefore, inhibition of the proliferation and migration of VSMCs is important for the prevention and treatment of cardiovascular complications in diabetes. Ras-related signaling induces the activation of the PI3K/Akt and extracellular signal-regulated kinase (ERK) signaling pathways to promote the proliferation and inflammation of VSMCs [6]. Recently, Yu et al. showed that the FAK-Src complex plays a molecular role in cell motility and facilitates cell migration [7]. Small GTPase is a key regulator of cytoskeletal organization, which regulates cell polarity and migration and affects different components of the cytoskeleton as well as cell-substrate adhesion and possibly matrix remodeling [8].

A previous study found that matrix metalloproteinase (MMP) increases metastasis and migration by damaging barriers formed by the extracellular matrix [9]. Matrix stiffness could modulate cell proliferation, invasion, and angiogenesis through several different mechanisms, including the ERK, FAK, and PI3K/Akt signaling pathways [10]. Nuclear factor-κB (NF-κB) has been reported to play a key role in MMP activation, particularly MMP2 and MMP9, which play a major role in the regulation of cell migration through autocrine or paracrine pathways [11]. A previous in vitro study suggested that in tumor cell lines, osteopontin stimulates MMP9 up-regulation via FAK, ERK, and NF-κB-dependent signaling pathways, which promote cytoskeletal organization, cell motility, and cell migration [12]. Furthermore, our previous study showed that diabetic conditions (OH) induced A7r5 cell migration and proliferation-related signaling and that acarbose treatment could potentially prevent atherosclerosis [13].

*Morus alba* L., generally called mulberry, is a genus of flowering plants belonging to the Moraceae family; it is extensively planted in China, Japan, and Korea [14]. The plant is composed of various phytochemicals, including anthocyanins, phenolic acids, flavonoids, and polyphenolic amides [15]. Previous studies confirmed that MLE can reduce inflammation and damage caused by non-alcoholic fatty liver and prevent hepatocarcinogenesis from inducing apoptosis and autophagy in our laboratory [16]. Mulberry has been known to modulate serum fasting glucose and lipids and to have anti-atherosclerotic properties [17]. Furthermore, nCGA was the most abundant polyphenol in mulberry leaves. Numerous data indicated that nCGA has antibacterial, antioxidant, and anticancer activities, especially hypoglycemic and hypolipidemic effects.

Our previous research used MLE and indicated that it could attenuate glucose-induced VSMC migration via inactivation of FAK and up-regulation of AMPK/RhoB signaling [18]. However, the mechanism by which components of mulberry leaf target small GTPase protein-mediated atherosclerosis remains unclear. In this study, we investigated the inhibitory effect of MLE and its major component, nCGA, in OH-induced VSMC migration and proliferation through several mechanisms, including the ERK, FAK, and PI3K/Akt signaling pathways. Furthermore, we also investigated its potential ability to prevent atherosclerosis.

## 2. Results

### 2.1. Effect of OH Medium, MLE and nCGA on Cell Viability and Proliferation of VSMCs

**Figure 1:**
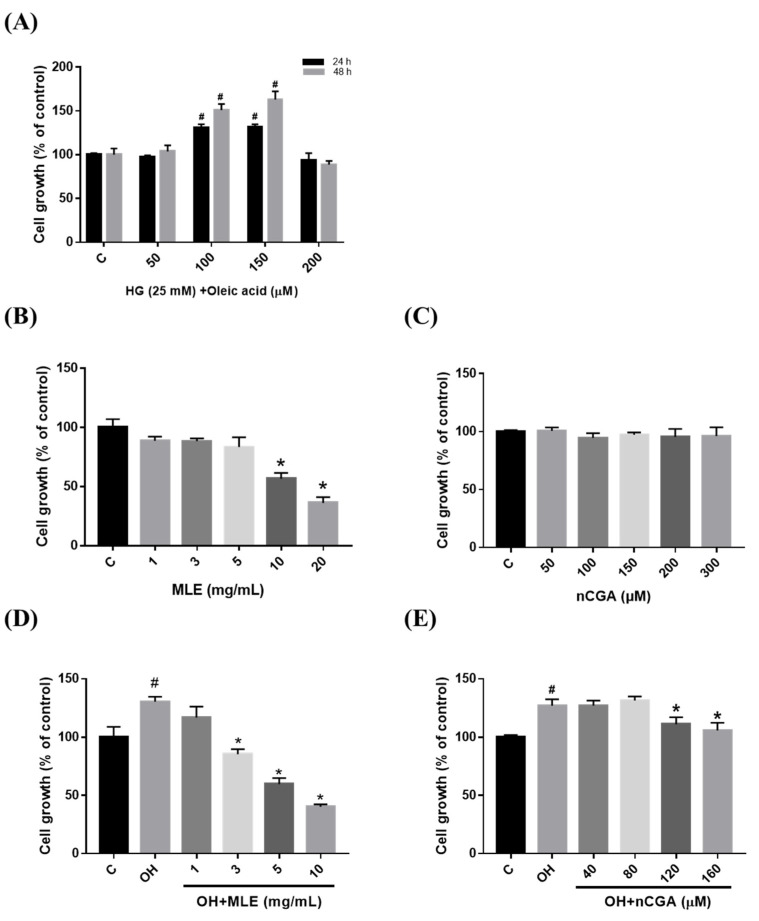
Effect of mulberry leaf extract (MLE) and neochlorogenic acid (nCGA) treatment on cell viability/proliferation. (**A**) A7r5 cell proliferation after being induced by HG (25 mM) and Oleic acid (0.5, 1.0, 1.5 and 2.0 mM) for 24 and 48 h. (**B**,**C**) A7r5 cells were treated with indicated concentrations of MLE (1.0, 3.0, 5.0, 10, and 20 mg/mL) and nCGA (0, 50, 100, 150, 200 and 300 μM) for 24 h. (**D**,**E**) A7r5 cells were pretreated with OH and then exposed to different concentrations of MLE (0.0, 1.0, 3.0, 5.0, and 10 mg/mL) and nCGA (0, 40, 80, 120, and 160 μM) for 24 h. Cell viability/proliferation of the A7r5 cells was evaluated using the 3-(4,5-dimethylthiazol-2-yl)-2,5 diphenyltetrazolium bromide (MTT) assay. The quantitative data are presented as the mean ± SD from a minimum of three independent experiments. # *p* < 0.05 as compared to the C (control). * *p* < 0.05 as compared to OH group.

We investigated the cytotoxicity of MLE and nCGA on VSMCs (A7r5 cell line). Initially, A7r5 cell proliferation was evaluated without cytotoxicity after 24 and 48 h of induction with high glucose (HG, 25 mM) and oleic acid (50, 100, 150 and the 200 µM), and the selected time point of 24 h treatment could promote the proliferation effect for subsequent experiments (Figure 1A). Then, the cell viability of A7r5 cells was assessed using indicate concentrations of MLE (1.0, 3.0, 5.0, 10 and 20 mg/mL) and nCGA (0, 50, 100, 150, 200, and 300 µM) for 24 h. Neither MLE (≤5 mg/mL) or nCGA (≤300 µM) treatment reduced cell viability in A7r5 cells (Figure 1B,C). Therefore, MLE (≤5 mg/mL) and nCGA (≤300 µM) were subsequently used to treat A7r5 cells.

In addition, we examine the suppressive effects of MLE and nCGA on the proliferation of VSMCs. A7r5 cells were pretreated with OH (150 μM oleic acid and 25 mM HG), and then exposed to different concentrations of nCGA (0, 40, 80, and 120 μM) for 24 h. The proliferation of A7r5 cells was inhibited by MLE at concentrations ≥ 3 mg/mL and nCGA at concentrations ≥ 120 μM (Figure 1D,E). These results revealed that the proliferation of A7r5 cells after OH was inhibited by MLE and nCGA.

### 2.2. MLE and nCGA Inhibit the Migration of VSMCs Cultured in OH

**Figure 2:**
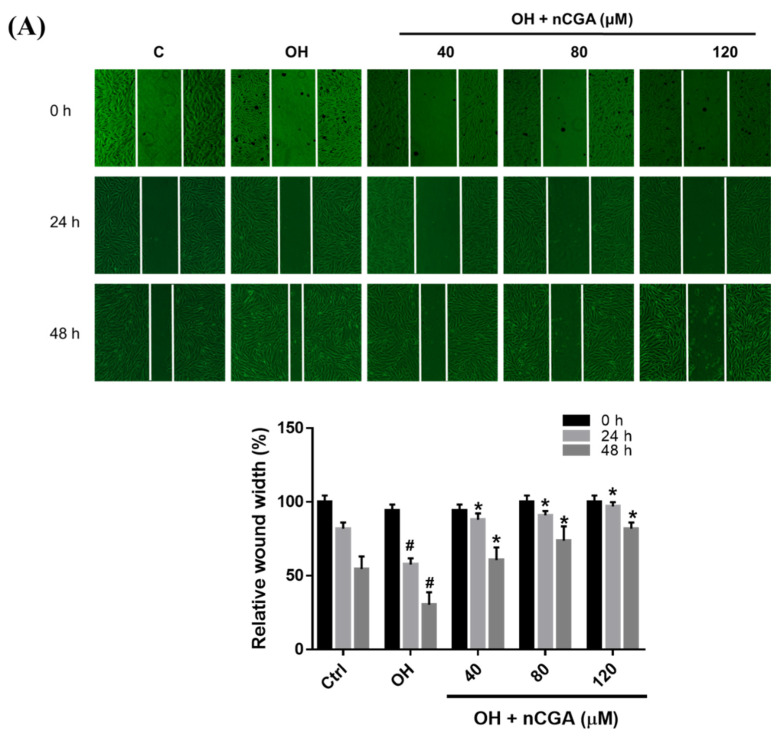

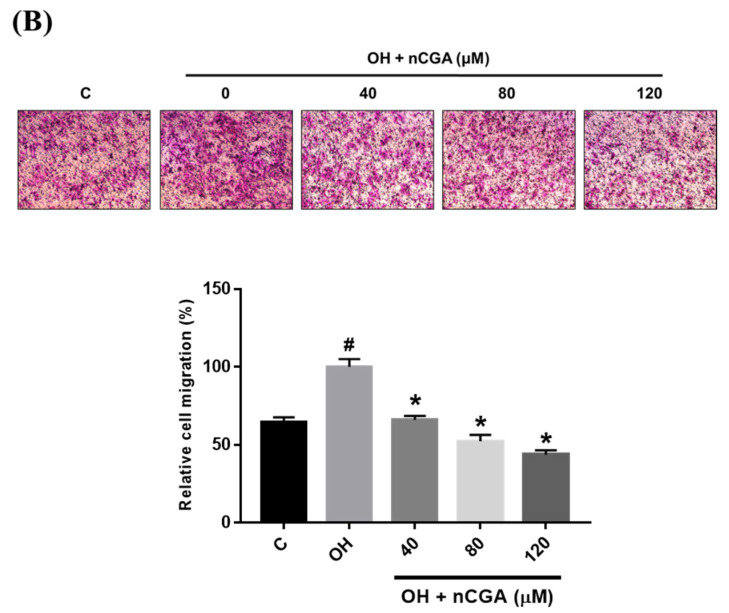
Effect of nCGA on the migration of OH cultured VSMCs. A7r5 cells were treated with OH and nCGA (0, 40, 80 and 120 μM) for 24–48 h. Migration was determined using the (**A**) Wound healing assay and (**B**) Transwell assay. The quantitative data are presented as the mean ± SD from a minimum of three independent experiments. # *p* < 0.05 as compared to the C (control). * *p* < 0.05 as compared to the OH group.

VSMC migration is a critical feature of the pathogenesis of atherosclerosis [19]. Therefore, the effect of nCGA on A7r5 cell migration under OH conditions was investigated. As exhibited in Figure 2A, A7r5 cells induced by the OH condition indicated the wound closure was increased. Notably, the results of wound healing were reduced by nCGA (40, 80 and 120 μM) compared with the OH group, indicating that nCGA decreased wound closure of cells in a time-dependent manner. Furthermore, we used the Transwell assay to investigate if cell migration was induced by OH. Taken together, these data demonstrated that the migration ability of cells under OH conditions was significantly reduced after treatment with nCGA (40, 80 and 120 μM) (Figure 2B).

### 2.3. MLE and nCGA Decrease the Expression of FAK-Related Signaling Proteins in OH Cultured VSMCs

**Figure 3:**
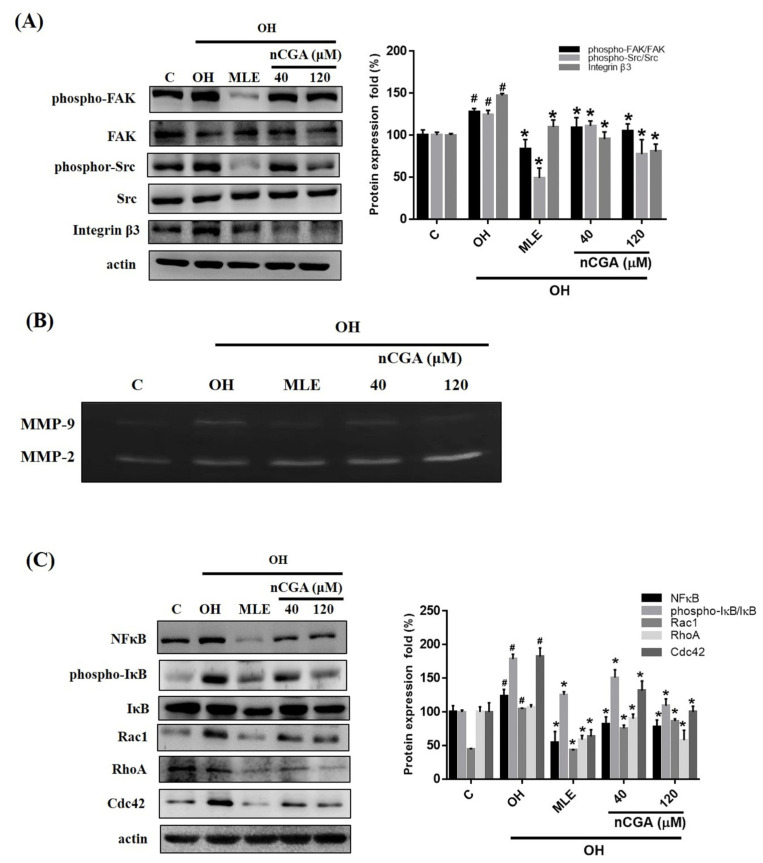
nCGA altered the migration-related proteins in OH cultured VSMCs. Cells were co-treated with OH, MLE, and nCGA (40 or 120 μM) for 24 h and then subjected to immunodetection conducted with antibodies against by Western blot analyses. (**A**) Densitometry was used to quantify p-FAK relative to total FAK, p-Src relative to total Src, and Integrin β3 relative to β-actin. (**B**) The gelatin zymography was then performed to analyze the activities of MMP-9 and MMP-2 as described in the Materials and Methods. (**C**) Densitometry was used to quantify NF-κB, p-IκB relative to total IκB, Rac1, Rho A, and Cdc42 relative to β-actin. The quantitative data are presented as the mean ± SD from a minimum of three independent experiments. # *p* < 0.05 as compared to the C (control). * *p* < 0.05 as compared to the OH group.

As the FAK-related signal is known to be a key regulator of cell migration in VSMC [20]. We investigated whether MLE and nCGA act through these signaling pathways to decrease OH-induced VSMC migration. A7r5 cells were co-treated with OH, MLE and nCGA (40 or 120 μM) for 24 h. The cells were then harvested, and Western blot analysis was conducted using antibodies against p-FAK, FAK, p-Src, Src, integrin β3, NF-κB, p-IκB, and small GTPase proteins Rac1, RhoA, Cdc42), and β-actin. The results revealed that MLE and nCGA treatment for 24 h reduced integrin β3 and p-Src expression and FAK phosphorylation (Figure 3A), the activities of MMP2, MMP9 (Figure 3B), and NF-κB, p-IκB expression, and small GTPases (Rac1, RhoA, and Cdc42) (Figure 3C) were also reduced in a dose-dependent manner, indicating that MLE and nCGA reduce migration-related signals in OH-induced A7r5 cells.

### 2.4. MLE and nCGA Inhibit Proliferation Related Proteins in OH Cultured VSMCs

**Figure 4:**
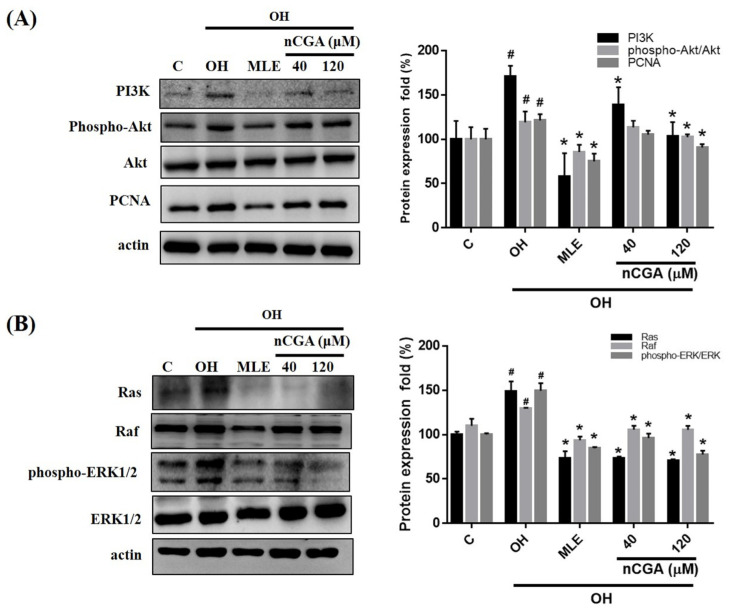
MLE and nCGA reduced the expression of proliferation-related proteins in diabetic cultured VSMCs. (**A**) The levels of PI3K, p-Akt, and PCNA in OH-treated A7r5 cells were notably increased under OH conditions and notably decreased after treatment with nCGA (40 and 120 μM). (**B**) In OH-stimulated A7r5 cells, the levels of Ras and p-ERK were increased, and OH-induced upregulation was nullified by nCGA. The quantitative data are presented as the mean ± SD from a minimum of three independent experiments. # *p* < 0.05 as compared to the C (control). * *p* < 0.05 as compared to the OH group.

PI3K/AKT and ERK pathway activation is mediated by the Ras protein and plays a critical role in cell proliferation [7,10]. We investigated whether MLE and nCGA act via the same pathway to inhibit OH-induced A7r5 cell proliferation. A7r5 cells were co-treated with OH and nCGA (40 and 120 μM) for 24 h. Western blotting revealed that the levels of PI3K, p-Akt, and PCNA were notably increased under OH conditions and notably decreased after treatment with nCGA (40 and 120 μM) (Figure 4A). In OH-stimulated A7r5 cells, the levels of Ras and p-ERK were increased, and OH-induced upregulation was abolished by nCGA treatment (Figure 4B). This demonstrated that MLA and nCGA suppress the expression of proliferation-related proteins in A7r5 cells.

### 2.5. MLE and nCGA Decreased Levels of OH-Induced ROS

**Figure 5:**
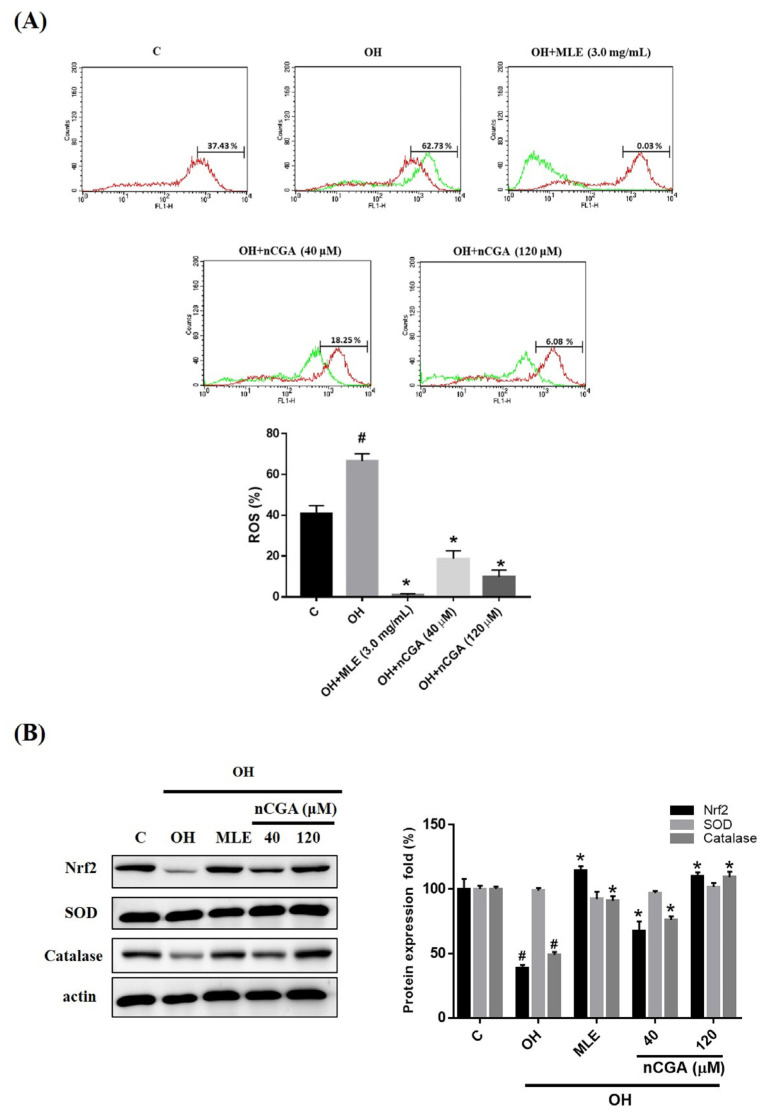
MLE and nCGA reduced intracellular reactive oxygen species (ROS) levels in OH cultured VSMCs. ROS levels in OH-treated A7r5 cells were increased and then notably decreased after treatment with MLE (3.0 mg/mL) and nCGA (40 and 120 μM). (**A**) ROS levels were measured by flow cytometry with a 10 μM carboxylated H2DCFDA analog (carboxy-H2DCFDA, C400). (**B**) The levels of Nrf2, SOD, and catalase in OH-treated A7r5 cells were notably increased under OH conditions and notably decreased after treatment with nCGA (40 and 120 μM). The quantitative data are presented as the mean ± SD from a minimum of three independent experiments. # *p* < 0.05 as compared to the C (control). * *p* < 0.05 as compared to the OH group.

Previous reports have indicated that ROS could induce cell proliferation and migration, and oleic acid or high glucose could elevate ROS levels [21]. Therefore, we examined the effect of OH on ROS levels in A7r5 cells using flow cytometry. As shown in Figure 5A, OH-induced metabolic conditions could significantly increase the generation of ROS compared with the control group. Furthermore, the levels of ROS in OH-treated A7r5 cells were decreased after treatment with MLE (3.0 mg/mL) and nCGA (40 and 120 μM). These results suggest that MLE and nCGA could inhibit the production of ROS in diabetic cultured cell lines. As shown in Figure 5B, MLE and nCGA increased the antioxidant enzyme-related proteins (Nrf2 and catalase). This study showed that MLE and nCGA can effectively decrease intracellular ROS levels and inhibit the proliferation and migration of VSMCs.

## 3. Discussion

MLE contains a lot of components, including quercetin, rutin, and chlorogenic acid, as well as many other antioxidants, which may be more effective than using nCGA alone to affect cell proliferation or migration-related proteins. It is used in food and pharmaceutical compounds due to its pharmacological effects, including being antioxidative, antibacterial, anticarcinogenic, hypoglycemic and hypolipidemic [22].

The previous study showed that nCGA was the most abundant polyphenol (approximately 0.355%) in MLE after HPLC and LC-MS analysis [23], and it possesses many health-promoting properties. An in vivo study of nCGA revealed that it significantly increased HDL-cholesterol, reduced total and LDL-cholesterol, and improved both the atherogenic index and cardiac risk factor [24]. In another study using an animal model, nCGA decreased hepatic steatosis and improved lipid profiles, glucose levels and insulin sensitivity [25]. In a previous clinical trial, nCGA-enriched instant coffee appears to have a significant effect on the absorption and utilization of glucose from the diet. This effect resulted in reduced body fat and body weight [26].

MLE has these medicinal effects and could potentially prevent atherosclerotic events through different pathways. A previous report by Yang et al. found that Mulberry leaf polyphenols have anti-atherogenesis effects via inhibition of LDL oxidation and foam cell formation in vitro experiments [27]. In addition, endothelial dysfunction parameters (Serum sVCAM-1: soluble vascular cell adhesion molecule-1, fibrinogen, and oxidized LDL), apolipoprotein A and apolipoprotein B were significantly (*p* < 0.001) reversed to near normal following treatment with MLE [28]. In our previous study, polyphenol-rich extracts from mulberry leaves inhibited VSMC proliferation, caused upregulation of p53 and inhibition of cyclin-dependent kinase [29]. Furthermore, MLE inhibits the development of atherosclerosis in cholesterol-fed rabbits and in cultured aortic VSMCs [30].

The proliferation and migration of VSMCs contribute to the pathogenesis and progression of atherosclerosis and cardiovascular disease. Growing evidence suggests that VSMC migration from the vascular media into the intima plays a crucial role in neointima formation in restenosis after angioplasty [30]. Previous studies have reported that angiotensin abrogates the inflammation, migration and proliferation of VSMCs via the inactivation of ROS-mediated PI3K/Akt and MAPK/ERK pathways [6]. In addition, insulin signal transduction also involves PI3K and Akt, which are the focus of current research on the molecular mechanism of insulin resistance [10]. Attenuating these signaling pathways might provide novel therapeutic options against insulin resistance and type 2 diabetes myelitis [31]. The present study was designed to determine the efficacy of MLE and nCGA for reducing VSMC proliferation and migration as well as the underlying mechanisms of atherosclerosis, which is the major cause of coronary artery disease.

Both the ERK and PI3K/Akt signaling pathways are important intracellular signaling pathways that play a key role in VSMC migration and proliferation during the formation of atherosclerotic plaques [7]. In the present study, we found that OH conditions markedly increased the phosphorylation of PI3K and Akt in VSMCs; however, this effect was then mediated by MLE and nCGA administration. Furthermore, treatment with either MLE or nCGA abolished the activation of ERK1/2, Rac1 and PCNA, as induced by OH. These results indicate that OH conditions may increase Ras activity and the subsequent activation of the PI3K/Akt and ERK/PCNA signaling pathways, which are involved in VSMC proliferation and migration responses; however, these effects can be inhibited by MLE and nCGA.

Another animal study revealed that nCGA protects cardiomyocytes from TNF-α–induced injury via inhibition of NF-κB signals, providing a novel therapeutic alternative for the prevention and treatment of heart failure [32]. Our previous study also found that MLE inhibited the migration of VSMCs through FAK/Src and NF-κB/small GTPase (RhoA, Cdc42, and Rac1) protein signaling pathways and further down-regulated the expression of MMP2, affecting F-actin cytoskeleton rearrangement [33].

The present study found that the increased phosphorylation of NF-κB/small GTPase and FAK/Src in VSMCs, as stimulated by OH, was effectively inhibited by MLE and nCGA. Treatment with either MLE or nCGA also attenuated the activation of Rac/RhoA, CDC42 and MMP2/9 as induced by OH. These results indicate that MLE and nCGA may play a crucial role in cardiovascular protection against OH conditions via suppression of VSMC migration through the FAK/Src and NF-κB/small GTPase protein pathways. On the other hand, this indicates that miR-143/145 are relevant to stress fiber formation and cytoskeleton. In the future, it can be verified whether nCGA can regulate miR-143/145 and affect the cytoskeleton.

Excessive ROS participates in the development of cardiovascular disease, VSMC hypertrophy and the apoptosis of endothelial cells [34]. Hyperlipidemia and hyperglycemia also induce ROS generation [35]. In this study, OH-induced metabolic conditions could promote the elevation of ROS levels and have been reported to decrease antioxidant status by flow cytometry assay. Co-treatment with MLE or nCGA could reduce the formation of ROS in OH-induced VSMCs and decrease VSMC injury and dysfunction. Our study showed the reduction in ROS levels in OH-induced VSMC (glucose effects) and increased antioxidant enzyme-related proteins (Nrf2 and catalase) when cells treat MLE or nCGA. We demonstrated that the ROS pathway is an important mechanism utilized by MLE or nCGA for reversing the OH-induced metabolic condition in A7r5 cells.

However, the present study shows that there are still some limitations to the clinical application of nCGA, such as the lack of in vivo studies. Therefore, future studies should use the diabetic db/db mice model to investigate the anti-atherosclerotic effects of mulberry leaf extract (MLE) and nCGA and to further clarify the pathogenesis. The development of nCGA as a new drug for the prevention and treatment of atherosclerosis requires improvements to its stability, solubility, and oral absolute bioavailability [36].

**Figure 6:**
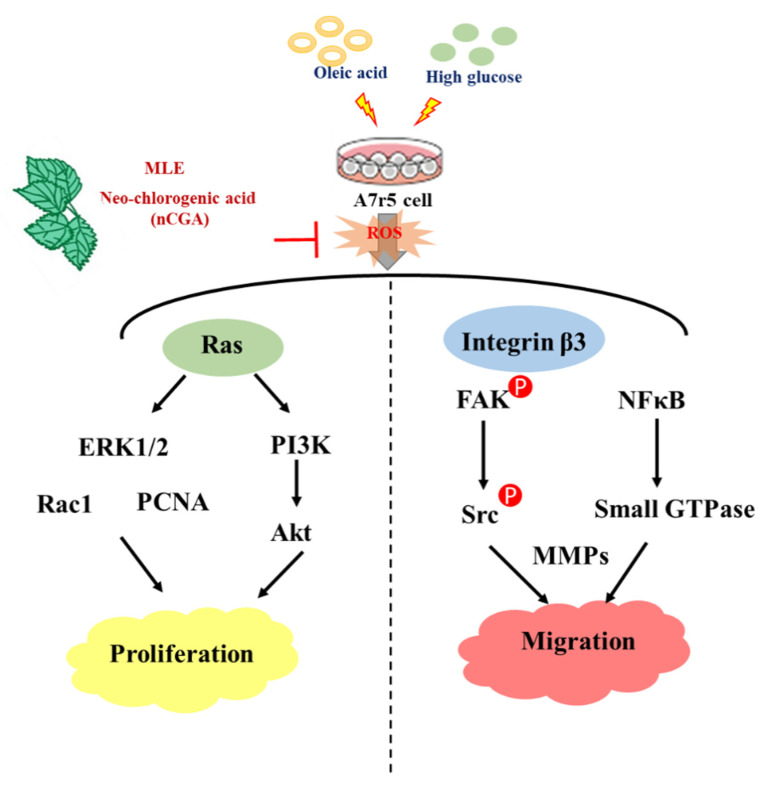
Schematic diagram of MLE and nCGA attenuation of VSMC proliferation and migration via inhibition of FAK and Ras-related signaling. ERK1/2, extracellular signal-regulated protein kinases 1 and 2; PI3K, phosphoinositide 3-kinases; Akt, protein kinase B; PCNA, proliferating cell nuclear antigen; FAK, focal adhesion kinase; MMPs, matrix metalloproteinases; NFκB, nuclear factor-κB.

In conclusion, nCGA is a natural product that can be obtained from MLE and has a wide pharmacological range. Compared with existing drugs, it has multisystem and multitarget pharmacological effects. We concluded that MLE and nCGA are indeed responsible for the observed positive effects of reduced VSMC migration and proliferation via inhibition of FAK/small GTPase proteins, PI3K/Akt and Ras-related signaling (Figure 6). Furthermore, they could potentially prevent atherosclerosis. nCGA may become a useful clinical drug for the treatment of metabolic syndrome and its related atherosclerotic lesion formation.

## 4. Materials and Methods

### 4.1. Chemicals and Reagents

Dulbecco’s modified Eagle’s medium (DMEM), fetal bovine serum (FBS), penicillin-streptomycin-amphotericin B (PSA), pyruvate, glutamine, and trypsin EDTA were obtained from Gibco (Grand Island, NY, USA) and Hyclone (Logan, UT, USA). The PVDF membranes, NC membranes, ammonium persulfate (APS), bisacrylamide, sodium dodecyl sulfate (SDS), and TEMED were acquired from Gibco (Grand Island, NY, USA) and Hyclone (Logan, UT, USA). Antibodies against PCNA (1:1000, NB100-456) and catalase (1:500, NBP2-24916) were acquired from Novus Biological (Littleton, CO, USA). PI3K (1:1000, #4292), NFkB (1:1000, #8242), phospho-FAK (1:1000, #3283), FAK (1:1000, #3285), phospho-Src (1:1000, #5473), Src (1:1000, #2108), Integrin β3 (1:1000, #4702), and SOD (1:500, #2770) were acquired from Cell Signaling Techonology (Beverly, MA, USA). Phospho-AKT (1:1000, ab133458), AKT (1:1000, ab8805), Ras (1:500, ab52939), Rac1 (1:500, ab97732), Cdc42 (1:1000, ab155940), and Nrf2 (1:500, ab137550) were purchased from Abcam (Cambridge, UK). RhoA (1:500, sc-418) was purchased from Santa Cruz Biotechnology (Santa Cruz, CA, USA). β-actin (1:10,000, A5441, as the internal control) and neochlorogenic acid (nCGA, purity > 98%) were purchased from Sigma-Aldrich (St. Louis, MO, USA). The nCGA was filtered using a 0.22 μm filter, and a 500 mM stock solution was prepared in dimethyl sulfoxide (DMSO) for further dilution in serial experiments. The final concentration of DMSO was not more than 1 mmol/L in each experimental group.

### 4.2. Cell Culture

Rat aortic vascular smooth muscle cells (VSMCs, A7r5) were purchased from the Bioresource Collection and Research Center (Food Industry Research and Development Institute, Hsinchu, Taiwan, cell number 60082). The cells were routinely cultured in DMEM supplemented with 10% FBS, 4.5 g/L glucose, 4 mmol/L L-glutamine, 1 mmol/L sodium pyruvate, 100 U/mL of penicillin, 100 μg/mL streptomycin, and 1.5 g/L sodium bicarbonate. The cells were cultured in an incubator containing 5% CO_2_ under a humid atmosphere at 37 °C.

### 4.3. Diabetic Conditions In Vitro Model and Experimental Design

Glucotoxicity is also known to be caused by oxidative stress and an increased risk of cardiovascular disease, stroke, and heart attack [37]. Therefore, we use 150 μM oleic acid and 25 mM high glucose (OH) to stimulate the production of glucotoxicity and a subsequent diabetic condition model (OH cultured VSMCs).

### 4.4. Preparation of MLE

Fresh mulberry leaves (obtained from Dadu Township, central Taiwan) were immediately dried and stored at room temperature. For MLE preparation, 100 g of dried mul-berry leaves was added into deionized water (3000 mL) and boiled at 95 °C for 3 h. The supernatants were filtered and concentrated through evaporation under reduced pressure at room temperature. The powders were stored at −80 °C temperature for the subsequent experiments. Then, the MLE powders were filtered using a 0.22 μm filter for further use in cell culture.

### 4.5. Cell Viability/Proliferation Assay

The MTT assay was used to measure cell viability/proliferation [38]. The A7r5 cells (5 × 10^4^ cells per well) were seeded in 24-well plates and incubated overnight with DMEM medium. A7r5 cells were treated with MLE and nCGA for 24 h. After treatment, 20 μL MTT (0.5 mg/mL) was added to each well and incubated for an additional 4 h at 37 °C. The supernatant was removed, and 200 μL isopropanol was used to dissolve a violet formazan complex, which was then shaken for 10 min. The absorbance at OD563 was measured with a spectrophotometric plate reader (Hitachi, Japan).

### 4.6. Wound Healing Assay

A7r5 cells were seeded into 6-well plate at a density of 1 × 10^6^ for 24 h and grown until 90–95% confluence. The medium used was serum starvation medium for 2 h before, and the monolayer of each well was straight-scratched using a 200 μL pipette tip. The unattached cells were then removed by washing with PBS, and the remaining cells were treated with OH. Then, the cells were treated with nCGA, and wounding was observed and photographed under a microscope at 0, 24 and 48 h. The images were used to calculate the wound area and average wound width by using the ImageJ software.

### 4.7. Transwell Migration Analysis

A7r5 cells were treated with nCGA and cultured with OH for 24 h. After treatment, cells were plated in the upper chamber and allowed to migrate to the lower chamber that contained 10% FBS for 12 h. The cells that had migrated into the lower surface of the insert were fixed and then stained with Giemsa solution (5%) for 60 min. Subsequently, the migrated cells were counted at magnification (400×) using a light microscope.

### 4.8. Western Blotting

Cell lysates were developed using RIPA buffer containing a phosphatase inhibitor and protease inhibitor cocktail at 4 °C for 1h. After being centrifuged at 10,000 rpm for 10 min, the supernatant was collected for the following analysis. Sample concentrations (50 µg) were separated into sodium dodecyl sulfate-polyacrylamide gel electrophoresis (SDS-PAGE) and transferred onto nitrocellulose membranes (Millipore, Bedford, MA, USA). The membranes were then blocked with 5% (*w*/*v*) non-fat milk powder/PBS at 37 °C for 1 h and then incubated with primary antibodies at 4 °C overnight. Thereafter, the membrane was washed with 0.1% Tween 20/PBS three times, and incubated with secondary antibodies (GE Healthcare, Little Chalfont, Buckinghamshire, UK) for 1 h after being washed with 0.1% Tween 20/PBS three times. Finally, the antibody–antigen complex was detected using enhanced chemiluminescence (ECL) reagent in FUJIFILM LAS-4000 (Tokyo, Japan). The resulting signals were acquired through densitometry using FUJFILM-Multi Gauge V2.2 software analysis system (Tokyo, Japan).

### 4.9. Gelatin Zymography

Gelatin zymography (MMP-2 and MMP-9) was performed as previously described [33]. A7r5 cells were cultured with OH and then treated with MLE (3 mg/mL) and nCGA (40 and 120 μM) in a humidified atmosphere of 5% CO_2_ for 24 h. A7r5 cells were seeded onto 6-well culture plates (5 × 10^5^ cells per well) and then starved with 1 mL of 0.5% DMEM for 24 h. After treatment, the culture medium was collected and centrifuged at 12,000 rpm for 5 min at 4 °C to remove cell debris. The sample was subjected to electrophoresis with 8% SDS-PAGE containing 0.1% gelatin. Following electrophoresis, the gels were washed twice with Triton X-100 (2.5%) on a gyrating shaker for 30 min incubated at room temperature to remove SDS. The gels were then incubated in reaction buffer (40 mM Tris-HCl, 10 mM CaCl_2_, and 0.01% NaN_3_) at 37 °C overnight. The gels were stained with Coomassie Brilliant Blue R-250 (0.1%) and then de-stained with deionized water. Gelatinase activity was displayed as horizontal white bands on a blue background.

### 4.10. Measurement of Intracellular Reactive Oxygen Species (ROS)

In order to investigate nCGA involvement in proliferation and migration, a 6-well plate was seeded with 7 × 10^4^ cells per well. After 24 h, the cells were treated with OH, or OH combined with MLE or nCGA at the indicated concentrations. ROS production was detected using the fluorescence probe 10 μmol/L carboxylated H2DCFDA analog (carboxy-H2DCFDA, C400). The cells were then incubated in the dark for 45 min, and the cellular ROS contents were detected by flow cytometry according to the manufacturer’s instructions. This nonfluorescent molecule is readily converted to a green-fluorescent form when the acetate groups are removed by intracellular esterase, and oxidation (by the activity of ROS) occurs within the cell.

### 4.11. Statistical Analysis

All experiments were performed at least three times. The results were expressed as the mean ± SD for each group. Statistical analysis was performed using Student’s *t*-test. A probability level of *p* < 0.05 was considered to indicate a statistically significant difference.

## Data Availability

Not applicable.
